# Neurological deterioration as a result of improper neck position detected by intraoperative neurophysiological monitoring in a cervical stenosis patient: A case report

**DOI:** 10.1097/MD.0000000000024241

**Published:** 2021-03-19

**Authors:** Tong Yu, Jiu-Ping Wu, Tao He, Yao-Kuan Ruan, Qin-Yi Liu

**Affiliations:** a Department of Spine Surgery, The Second Hospital of Jilin University; b College of Clinical Medicine, Jilin University, Changchun, Jilin Province, China.

**Keywords:** ACDF, cervical stenosis, intraoperative, monitoring, neurophysiological

## Abstract

**Rationale:**

Intraoperative neurophysiological monitoring (IONM) is widely used in spinal surgeries to prevent iatrogenic spinal cord injury (SCI). Most surgeons focus on avoiding neurological compromise intraoperatively, while ignoring the possibility of nerve damage preoperatively, such as neck positioning. Thus, this study aims to report a case with transient neurological deterioration due to improper neck position detected by IONM during cervical surgery.

**Patient concerns:**

A 63-year-old male patient had been suffering from hypoesthesia of the upper and lower extremities for three years.

**Diagnoses:**

Severe cervical stenosis (C5-C7) and cervical ossification of a posterior longitudinal ligament.

**Interventions:**

The cervical stenosis patient underwent an anterior cervical corpectomy decompression and fusion (ACDF) surgery with the assistance of IONM. When the lesion segment was exposed, the SSEP and MEP suddenly elicited difficulty indicating that the patient may have developed SCI. All the technical causes of IONM events were eliminated, and the surgeon suspended operation immediately and suspected that the IONM alerts were caused by cervical SCI due to the improper position of the neck. Subsequently, the surgeon repositioned the neck of the patient by using a thinner shoulders pad.

**Outcomes:**

At the end of the operation, the MEP and SSEP signals gradually returned to 75% and 80% of the baseline, respectively. Postoperatively, the muscle strength of bilateral biceps decreased from grade IV to grade III. Besides, the sensory disturbance of both upper extremities aggravated. However, the muscle power and hypoesthesia were significantly improved after three months of neurotrophic therapy and rehabilitation training, and no complications of nerve injury were found at the last follow-up visit.

**Lessons:**

IONM, consisting of SSEP and MEP, should be applied throughout ACDF surgery from the neck positioning to suture incisions. Besides, in the ward 1to 2 days before operation, it is necessary for conscious patients with severe cervical stenosis to simulate the intraoperative neck position. If the conscious patients present signs of nerve damage, they can adjust the neck position immediately until the neurological symptoms relieve. Therefore, intraoperatively, the unconscious patient can be placed in a neck position that was confirmed preoperatively to prevent SCI.

## 1 Introduction

ACDF, as a conventional technique of anterior cervical spine surgery, is an effective method to treat cervical spine diseases.^[1–3]^ Due to updated imaging technologies, improved surgical procedures, and the aging population, the number of cervical surgeries has been uninterruptedly increasing.^[4]^ Surgical complications of anterior cervical spinal surgery include cervical SCI, nerve root impairment, vascular compromise, prevertebral hematoma, swallowing dysfunction, esophageal damage, and wound infection.^[5,6]^ According to previous studies, the occurrence of iatrogenic neurological injury ranges from 3.2% to 0.3% after cervical spinal surgeries.^[7–9]^ In spite of an infrequent incidence, iatrogenic neurological deterioration may result in irreversible consequences. Thus, it is comparatively necessary for spinal surgeons to clearly know the real-time feedback of the neural functional state during operation.

In 1979, Brown et al.^[10]^ first reported the use of IONM to reduce the risk of scoliosis surgery. In 1988, Meyer et al.^[11]^ described SSEP-assisted spinal cord surgery, which reduced the incidence of neurological damage from 6.8% to 0.7%. In 1993, Epstein et al.^[12]^ found in 100 cervical spine surgeries that SSEP reduced quadriplegia from 3.7% to 0%. However, neurological deterioration and even quadriplegia were found postoperatively, although there were no alerts from SSEP intraoperatively.^[11]^ This was because SSEP can only reflect the function of the dorsal spinal cord, so SSEP pointed out that there was no abnormality when the ventral spinal pathway was damaged.^[13–17]^ Subsequently, MEP was used to monitor the ventral spinal pathway, which perfectly compensated for the previous limitation.^[18]^ Li et al.^[19]^ suggested that the sensitivity of combined MEP and SSEP in cervical spine surgeries can reach 100%. Consequently, the combined use of SSEP and MEP in spinal surgery has been advocated by many scholars.^[4,14,20,21]^

To prevent iatrogenic SCI, IONM was widely used in cervical spinal surgeries during the critical phases of the surgical procedure.^[19,22–27]^ Most surgeons only focus on avoiding neurological injury during the operation. However, they ignored the possibility of nerve damage preoperatively, such as neck positioning. Therefore, we present a case with transient neurological deterioration as a result of improper neck position detected by IONM during ACDF for the management of severe cervical stenosis.

## 2 Material and methods

### 2.1 Ethics

Ethics approval for this study was obtained from the Ethics Committee of The Second Hospital of Jilin University. The patient provided written informed consent for this report, and his information has been anonymous.

## 3 Case report

### 3.1 Patients

**Figure 1:**
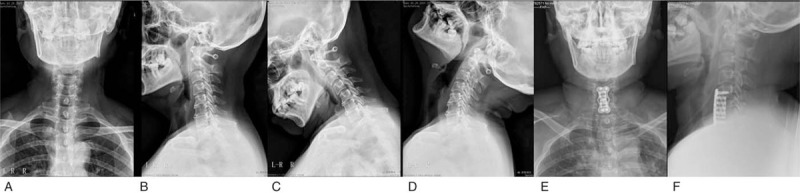
Radiographs of the cervical spine showed decreased cervical curvature, hyperosteogenesis at the edge of the C5, C6 and C7, and narrowing intervertebral space of the C5-C6 and C6-7. A, B represents the anteroposterior and lateral position respectively. C, D indicates the dynamic position. E, F shows a good position of the internal fixation.

**Figure 2:**
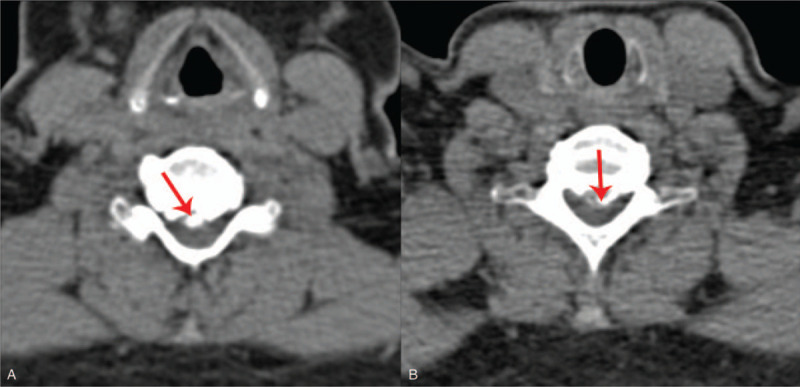
CT images revealed deflation of physiological curvature of the cervical spine, hyperosteogenesis of multiple cervical vertebrae, and strip-shaped high-density shadows with spinal stenosis in the C5-C7 segment (red arrow).

**Figure 3:**
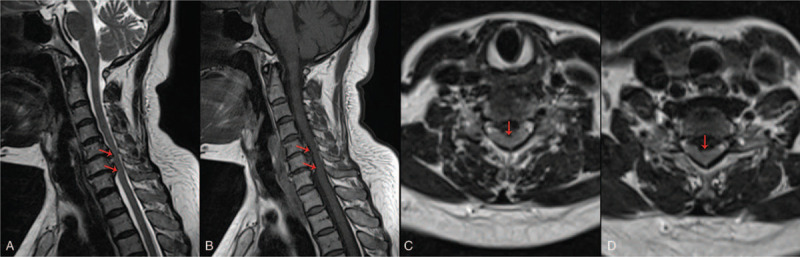
MRI showed spinal stenosis at the C5-C7 segment, C5-C6 and C6-7 cervical disc herniation and compression of the dura (red arrow). Besides, there were no abnormal signals in the cervical spinal cord.

A 63-year-old male patient had been suffering from hypoesthesia of extremities for three years. Preoperative radiographs showed severe cervical stenosis and spinal cord compression from C5 to C7 (Figs. 1–3). Physical examination results are as follows: the hypoesthesia was distributed in the left arm, right hand, trunk, and lower limbs. The muscle strength of bilateral deltoid, bilateral biceps, bilateral triceps, and bilateral fissure, was grade IV. The power of bilateral iliopsoas muscle, bilateral quadriceps femoris, and bilateral tibial anterior muscle were all grade V. Bilateral knee tendon reflexes and Achilles tendon reflexes were hyperactive. Bilateral Hoffman and Babinski signs were positive. Lasegue test and Bragard sign of bilateral lower extremities were negative. He was diagnosed with severe cervical stenosis (C5-C7) and cervical ossification of the posterior longitudinal ligament.

### 3.2 Anesthesia

The patients received ACDF surgery in a supine position. Total intravenous anesthesia was carried out with the standardized protocol. An intravenous loading dose of Remifentanyl 75 mg/kg (Remifentanil Hydrochloride for Injection, National pharmaceutical group industry co. LTD. Langfang branch. Hebei, China) and Propofol 3 mg/kg (Propofol Injection, Guangdong Jiabo pharmaceutical co. LTD. Chian) were used for the induction. Additionally, Propofol 2to 5 mg/kg per hour and Remifentanyl 100 mg/ml according to the weight table were continually infused for maintaining anesthesia. We did not use muscle relaxant or inhalant for induction after intubation to reduce the interference factors of IONM.

### 3.3 Neurophysiological monitoring

**Figure 4:**
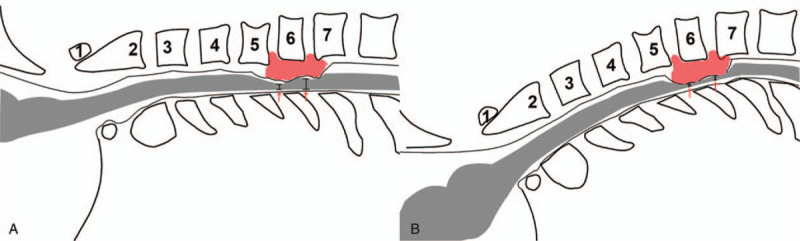
The example of the patient's (A) before and (B) after neck positioning on the operative table. The diameter of the spinal cord in figure A was significantly larger than that in figure B (red arrow).

The baseline was recorded before (Fig. 4A) and after (Fig. 4B) neck positioning but before surgical manipulation. Intraoperatively, there was frequent communication between the surgeon, anesthesiologist, and neurophysiologist to determine the functional state of the spinal cord. SSEP latency prolonged by more than 10%, or SSEP signal amplitude decreased by more than 50%, was defined as long-term monitoring alarm. The amplitude of MEP decreased by more than 50% was classified as IONM alerts.

We utilized the Nicolet Endeavor CR^TM^ IONM system (Nicolet, 5225 Verona Road, Bldg. 2 • Madison, Wisconsin 53711-4495, USA), and disposable subdermal needle electrodes (Guangzhou Nicolet Scientific Instrument Co., Ltd, China), which were 0.4 mm in diameter and 12 mm in length. For IONM, a standard scheme, including SSEP and MEP, was conducted. The MEP was recorded from the disposable subdermal needle electrodes inserted into muscles, including the bilateral biceps, bilateral abductor pollicis, bilateral abductor halluces, bilateral gastrocnemius, bilateral peroneal long muscle, and bilateral vastus lateralis. MEP stimulation needle electrodes were inserted into the scalp at C1 and C2, respectively. The stimulation intensity was 360 V. Meanwhile, the recording disposable subdermal needle electrodes of SSEP were inserted at C3 and C4 of the scalp, and with Cz referring to Fpz. The stimulation needle electrodes of SSEP were placed on the superficial position of the median nerve and the posterior tibial nerve. The intensity was 30 mA, the duration was 300 μs, and the rate was 4.1 Hz.

### 3.4 Clinical outcomes and follow-up

**Figure 5:**
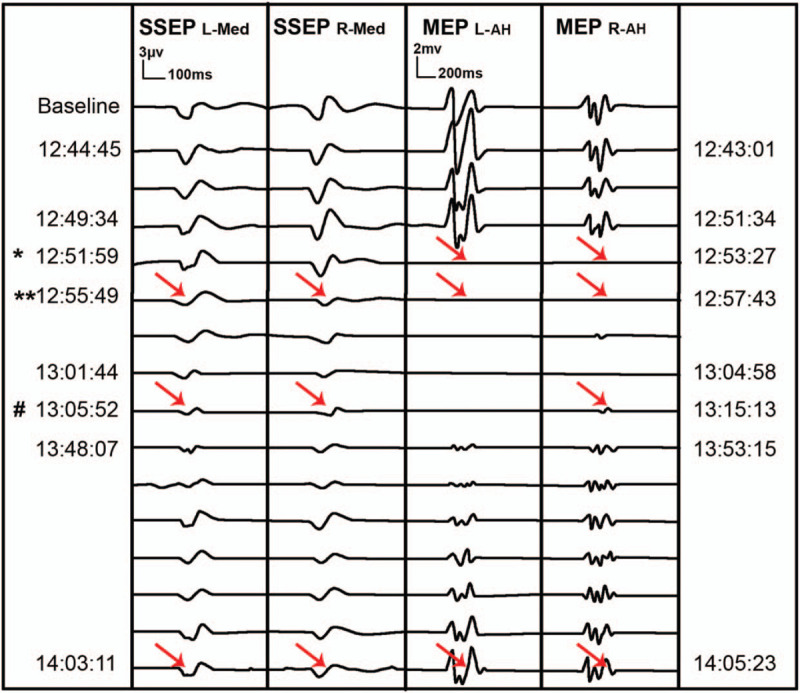
SSEP and MEP signals were obtained intraoperatively. ∗ After neck positioning, we found that the MEP of bilateral abductor halluces caused the SSEP of the bilateral median nerve to decrease significantly (red arrow). ∗∗ The surgeon suspended the operation and immedicably repositioned the neck of this patient (red arrow). ^#^ The MEP of the right abductor halluces muscle showed signs of recovery, but the amplitude was relatively short (red arrow). AH= abductor halluces, L= left, Med= median nerve, R= right.

The 63-year-old male underwent an ACDF surgery with the assistance of IONM for the treatment of severe cervical stenosis. When the lesion segment was exposed intraoperatively, the SSEP and MEP suddenly elicited difficulty (Fig. 5). All the technical causes of IONM events were eliminated, and the surgeon suspended operation immediately and suspected that the IONM alerts were caused by cervical SCI due to the improper position of the neck. Subsequently, the surgeon repositioned the neck of the patient by using a thinner shoulders pad. At the end of the operation, the MEP and SSEP signals gradually returned to 75% and 80% of the baseline level, respectively (Fig. 5). Postoperatively, the muscle strength of bilateral biceps decreased from grade IV to grade III. Besides, the sensory disturbance of both upper extremities aggravated. However, the muscle power and hypoesthesia were significantly improved after three months of neurotrophic therapy and rehabilitation training, and no complications were found at the last follow-up visit. The follow-up period lasted for 4 years.

## 4 Discussion

IONM has the advantage of real-time feedback on the integrity of spinal pathways,^[13–16,19,22–29]^ which is a relatively indispensable element in ACDF surgery for severe cervical stenosis. Currently, it is routinely used in a variety of spine surgeries, including tumor resection,^[16,29–32]^ deformity surgery,^[13,28,33,34]^ trauma,^[14]^ and degenerative spine surgery.^[35–37]^ Neurological injuries can be caused by many risk factors, such as traction, compression, or ischemia of the spinal cord, and can be detected by IONM.^[15,20,21,28,29,38–41]^ However, cervical SCI induced by improper neck position and early detected by IONM has been rarely reported. Thus, we describe a case with transient neurological deterioration as a result of improper neck position detected by IONM during ACDF surgery for a severe cervical stenosis patient.

The neck position is closely related to the success of ACDF surgery. A good position of the neck could be beneficial to the exposure of the surgical field and avoid excessive traction of the esophagus, trachea, and adjacent soft tissues. Therefore, the neck positioning of anterior cervical surgery is also a crucial surgical phase. In general, surgeons put the patient in a supine position, place a shoulders pad under the shoulders of the patient to raise the shoulders by about 20° , lean the head back, and then set around pillow under the neck to prevent it from hanging. In the present study, we believed that the improper neck position induced the neurological deterioration in this severe cervical stenosis patient and suggested that IONM is relatively indispensable for ACDF surgery, especially in patients with severe cervical stenosis. In addition, preoperatively, we encourage patients to imitate the neck posture during operation and observe whether the patient can tolerate this posture.

According to previous studies, the authors suggest that the position of the patient on the operating table is closely related to vascular occlusion in the upper and lower extremities.^[42–44]^ To our knowledge, three reports in the studies described unilateral or bilateral femoral artery occlusion due to improper position, all of which were detected by IONM.^[33,43,44]^ Trammell et al.^[45]^ recommended that the reversible and safety ischemia duration of the lower extremities should not exceed three hours. In the present study, cervical SCI was induced by the improper neck position intraoperatively. Fortunately, the inferior function of the spinal cord was detected by IONM in time, which alerted the surgeon to take remedial measures as soon as possible. Consequently, catastrophic and irreversible neurological impairment can be prevented. We attribute this positive clinical outcome to IONM. Besides, we advocate the use of IONM throughout the cervical spinal surgery, and this viewpoint was in accordance with Forster et al ^[31]^

In our study, to diminish the incidence of false-negative and false-positive IONM alerts,^[46]^ we used SSEP and MEP modalities to reveal the real-time feedback on the integrity of dorsal and ventral spinal pathways. The amplitude of both SSEP and MEP of the patient decreased by more than 50%. After eliminated the following technical factors, including neuromuscular blocking agents, hypoxemia, hypoperfusion, hypothermia, hypotension, and blood rheology,^[9,47–54]^ the surgeon suspected that the IONM alerts might be caused by SCI and immediately suspended the operation, and then removed the shoulder pad. Other measures recommended in the literature, such as the increase of mean arterial pressure and intravenous methylprednisolone pulse treatment, were also performed.^[13,14]^ Subsequently, the surgeon repositioned the neck of patient and completed the operation with a thinner shoulders pad. The SSEP and MEP were gradually restored intraoperatively, but neither SSEP nor MEP returned to the baseline before the incision was closed. In this study, the patient experienced neurologic complication postoperatively, manifested as bilateral biceps dropping from level 4 to level 3, and radiative pain and numbness in both upper extremities. However, muscle strength and hypoesthesia were significantly improved after three months of neurotrophic therapy and rehabilitation training. We consider that it is quite essential to use IONM in ACDF surgery. In other words, if IONM was not applied throughout the surgery, this patient may have suffered from irreversible SCI.

The authors^[55,56]^ reported that early decompression is favorable to the recovery of SCI. Thus, in the present study, after confirming the deterioration of spinal cord function, the surgeon completed anterior cervical spinal decompression as soon as possible. Hence, we could avoid irreversible SCI in this patient, which might be attributed to the accuracy and early IONM alerts.

## 5 Conclusions

IONM could promptly alert impending SCI in ACDF surgery at the early stage. IONM, consisting of SSEP and MEP, should be applied throughout ACDF surgery from the neck positioning to suture incisions. Besides, in the ward 1–2 days before operation, it is necessary for conscious patients with severe cervical stenosis to simulate the intraoperative neck position. If the conscious patients present signs of nerve damage, they can adjust the neck position immediately until the neurological symptoms relieve. Therefore, intraoperatively, the unconscious patient can be placed in a neck position that was confirmed preoperatively to prevent SCI.

## Acknowledgments

At present, the doctor-patient relationship in China is relatively poor. Post-operatively, the patient encountered surgical complications. However, he still had full confidence in our treatment technology, actively cooperating with the rehabilitation treatment, and achieved a satisfactory treatment effect finally. We are very grateful to our patients for their trust, support, and recognition.

## Author contributions

**Data curation:** Tao He.

**Methodology:** Tong Yu, Jiuping Wu.

**Project administration:** Jiuping Wu, Yaokuan Ruan.

**Software:** Tao He, Yaokuan Ruan.

**Supervision:** Qinyi Liu.

**Validation:** Qinyi Liu.

**Writing – original draft:** Tong Yu.

**Writing – review & editing:** Qinyi Liu.
